# Use of Time-Frequency Representation of Magnetic Barkhausen Noise for Evaluation of Easy Magnetization Axis of Grain-Oriented Steel

**DOI:** 10.3390/ma13153390

**Published:** 2020-07-31

**Authors:** Michal Maciusowicz, Grzegorz Psuj

**Affiliations:** Department of Electrical and Computer Engineering, Faculty of Electrical Engineering, West Pomeranian University of Technology, ul. Sikorskiego 37, 70-313 Szczecin, Poland

**Keywords:** non-destructive testing, magnetic Barkhausen noise, magnetic anisotropy, grain oriented steel, time-frequency representation, signal processing, data mining methods

## Abstract

The paper presents a new approach to non-destructive evaluation of easy/hard magnetization axis in grain-oriented SiFe electrical steels based on the Barkhausen phenomenon and its time-frequency (*TF*) characteristics. Anisotropy in steels is influenced by a number of factors that formulate the global relationship and affect the Barkhausen effect. Due to the observed high variability in the dynamics of magnetic Barkhausen noise (MBN) over time, obtained for various directions in grain-oriented steel, it becomes justified to conduct MBN signal analyses in the time-frequency domain. This representation allows not only global information from MBN signal over entire period to be expressed, but also detailed relationships between properties in time and in frequency to be observed as well. This creates the opportunity to supplement the information obtained. The main aspect considered in the work is to present a procedure that allows an assessment of the resultant angular characteristics in steel. For this purpose, a sample of a conventional grain-oriented SiFe sheet was used. Measurements were made for several angular settings towards the rolling and transverse directions. A data transformation procedure based on short-time Fourier transform (STFT) as well as quantitative analysis and synthesis of information contained in the *TF* space was presented. Angular characteristics of selected *TF* parameters were shown and discussed. In addition, an analysis of the repeatability of information obtained using the proposed procedure under various measurement conditions was carried out. The relationship between the selection of calculation parameters used during transformation and the repeatability of the obtained *TF* distributions were demonstrated. Then the selection of the final values of the calculation parameters was commented upon. Finally, the conclusions of the work carried out were discussed.

## 1. Introduction

Magnetic Barkhausen noise (MBN) is a phenomenon that occurs when a ferromagnetic material is being magnetized. During that process, when the magnetic field strength increases, the magnetic domain structure is gradually reorganized. This process is associated with the movement of domain walls (DW), followed by the rotation of magnetization vectors inside domains, resulting in discontinuous changes in the magnetic flux inside a material [1,2,3]. Those changes in the magnetic flux can be observed by a coil located at the surface of the material, in the form of voltage noise induced in its turn as a result of rapid changes in the degree of magnetization [4]. This method is used for various industrial applications for non-destructive condition testing and quality assessment of ferromagnetic materials, e.g., for evaluation of residual stress, active tensile and compressive stress, microstructure and micro hardening [5,6,7,8,9,10,11,12,13,14,15,16,17,18,19]. In addition to the possible applications mentioned above, the MBN is also used to evaluate the directional properties of ferromagnetic materials. Within this group of applications, an important aspect is the assessment of magnetic anisotropy of materials and the detection of easy magnetization axes. Recently, the use of MBN to evaluate magnetic anisotropy in steels, which is the result of a number of different factors, has become an equally important issue. The key in this aspect is the possibility of non-destructive and quick assessment of directional magnetic characteristics (introducing the axis of easy/hard magnetization) of steel formulated specially by means of surface engineering methods.

Determining the axis of easy magnetization is particularly important in the process of designing electrical machines, in essence the transformer cores or electric motors. Anisotropic properties allow optimizing their constructions and in consequence increasing effectiveness and reduce the costs of transformation and distribution of energy [20,21]. The resultant easy magnetization axis is influenced by a number of factors, including: residual stress, grain shape and size, etc. [22,23]. Depending on the influence of each factor, the value of anisotropy will change, which changes the magnetization ability in different directions. Within the currently conducted research on the application of the MBN to assess the magnetic anisotropy of materials, two approaches can be distinguished. The first one is related to the analysis of the impact of individual factors and its rate on the scale of anisotropy [24,25,26]. The second one refers to possible application for the need of rapid assessment of the resultant axis of easy magnetization [27,28]. This paper considers the latter aspect.

In the literature, different approaches to assessing the easy magnetization axis can be distinguished by omnidirectional observation of MBN activity. The following description presents several literature examples of the use of MBN to detect the easy magnetization axis. The first, classic approach is to observe the MBN for various settings of directional magnetic field acting on the examined material. In paper [28], the MBN burst expressed in the time domain was analyzed to obtain information about anisotropic properties. A classical grain-oriented electrical steel sheet was used for the study. The authors observed a higher amplitude value of MBN signal envelope for the rolling direction (RD), which was characterized by the occurrence of two distinct peaks in comparison to the signals obtained for other directions. They explained this phenomenon with much higher MBN activity for RD due to the large number of 180° domain walls (DWs), and associated the maximum values with the process of nucleation and annihilation of DWs. Confirmation of these observations was a distribution of the root-mean-square (RMS) value of the measured MBN signal versus the measuring angle, showing the highest value along the RD direction. An attempt to overcome the need for mechanical rotation of the measuring head is to introduce an appropriate modification of the MBN transducer’s excitation system. Examples of applications of such original solutions were presented in papers [27,29]. In [29], the authors used two C-shaped cores with coils placed perpendicularly to each other to control the excitation characteristics, a method named rotational Barkhausen noise (RBN). In this way, it is possible to generate a two-dimensional magnetizing field imitating the characteristics occurring during the classical MBN angular measurement, without the need for mechanical rotation of the magnetizing system. A disadvantage of this approach is the problem to obtain full homogeneity of the magnetic field. In paper [27], a rotatable transducer was used, in which the rotating permanent magnets generated a magnetic field, and the centrally located coil measured the MBN signal. The transducer collected data of the generated in the material magnetic induction disturbances, and then the measured MBN signal was analyzed in the time domain using features extracted from the MBN signal’s envelope. This method, referred to as continuous rotational Barkhausen noise (CRBN), allows straight observation of directions for which there is a clear increase in magnetic emission. The directional characteristics were related to the anisotropy of the material. The performance of the proposed system was verified based on the comparison with the classical approach. Despite the high correlation between the results obtained and those of the classic angular measurement, the key issue of the proposed CRBN method is the selection of the speed of the transducer motion and the rotation of the magnets. Both factors have a large impact on the sensitivity of the measurement and, as a result, the discrimination between the directional magnetic properties of the material (directions of magnetization). Mindful of the need to increase the effectiveness of assessing the anisotropic properties of magnetic materials, the idea of sequential analysis of the MBN burst has recently appeared. Its purpose is to divide a single burst into bands (sections) defined in relation to the magnetization period or magnetic field level. An example of such an approach was presented in [25,30,31]. In those works, the area of greatest MBN activity (referred to main MBN peak) was associated by authors with the motion of 180° DWs referring to the effect of roll magnetic anisotropy (RMA). The two other sub-periods occurring before and after the major one were associated, respectively, with the process of nucleation of reverse domains influenced by magneto-crystalline anisotropy (MCA) energy and motion of 90° DWs. In that work, from each section the energy value of MBN was calculated, and then the angular characteristics of the energy for individual sub-periods were achieved. The results obtained confirmed the possibility of a more accurate analysis of the phenomenon of anisotropy. However, the main problem of the method is the appropriate determination of signal divisions into sub-periods. Additionally subsequent interpretation requires consideration of many factors. Regardless of the approach finally applied, the named factors that define anisotropy are not the only ones that affect the course of angular characteristics of the MBN. The measurement conditions also have a significant impact on the effectiveness of anisotropy detection and measurement sensitivity, as presented and discussed in [31]. Similar observations were made in [23], where the need to maintain a constant value of magnetic flux density was underlined. It was also stated that during the tests it is necessary to observe not only the height of the MBN envelope but also its shape. All of the above factors result in a requirement for a broad analysis of the dynamics of the observed Barkhausen phenomenon, including both time and frequency characteristics. This entails the need for sequential monitoring of time and frequency characteristics in successive short time windows. The synchronous approach creates the opportunity to supplement the acquired information. Therefore, it becomes justified to carry out time-frequency (*TF*) analysis of measured MBN signals. This way of presentation allows more detailed information about the analyzed phenomenon to be obtained, because in addition to the extraction of parameters integrating global information (such as rms, energy or number of pulses) about the signal within the entire MBN period, it is possible to observe detailed relationships between properties in time and in frequency for subsequent time moments. As a result, it is possible to perform additional analysis and provide more detailed data on the nature of the phenomenon, which may be important for carrying out the final assessment. Recently, the authors presented the results of works on the application of the *TF* transformation to quantify the MBN signal information [24,32]. The [32] presents a broad description of the proposed *TF* analysis, *TF* features extraction and their interpretation along with a comparison of the information they carry with respect to the classic features of the MBN signal determined in the time or frequency domain. The influence of measuring conditions on the discrimination level was also discussed. The second paper of the authors [24] proposes a procedure based on *TF* characteristics enabling a clear division of the MBN signal into the aforementioned three sub-bands and determining the corresponding distributions of directional characteristics. However the described procedure did not allow the global anisotropy characteristic to be defined, being an assembly of all factors. Therefore, this paper proposes a procedure for determining the resultant easy magnetization axis based on the *TF* representation as well as a detailed analysis of the factors affecting the results. The successive sections present a setup of the measuring system, a proposed procedure for analyzing and evaluating the resultant easy magnetization axis. Ultimately, the results will be discussed and conclusions will be presented.

## 2. Samples and Measuring System Setup

**Figure 1:**
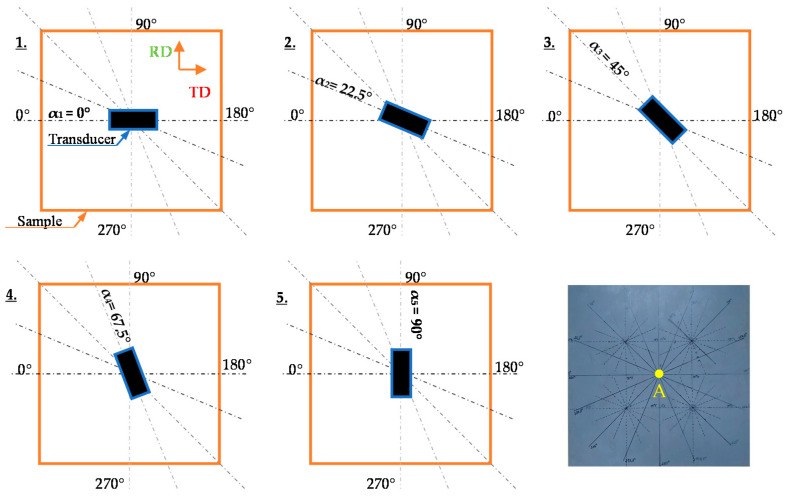
Measurement steps and view of conventional electrical steel sheet with marked measurement location.

**Figure 2:**
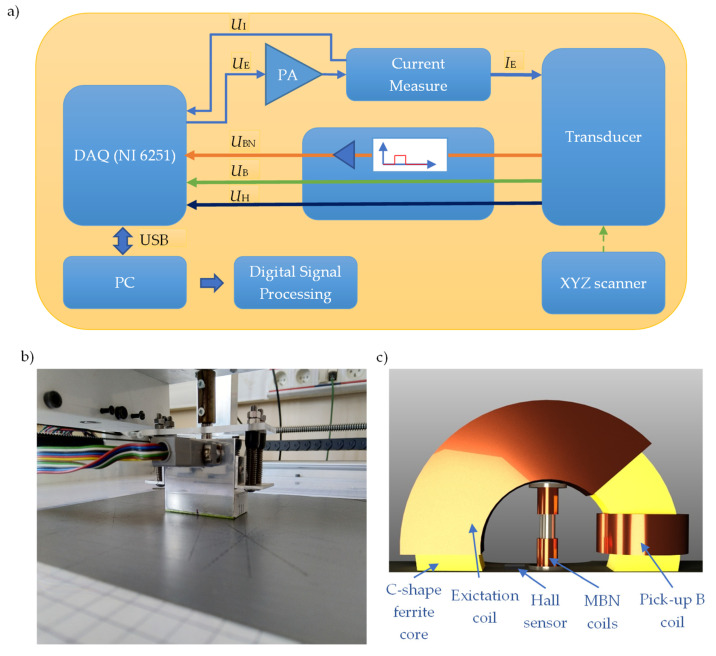
The measurement system and XYZ scanner with mounted transducer: (**a**) schematic view of the configuration, (**b**) photo and (**c**) 3D view of the transducer.

In the experiment, the conventional cold-rolled 3%SiFe electrical steel sheet (in reference to EN 10107 and IEC 60404-8-7 Standards) of 0.27 mm thickness, with silicon contents of 3% by mass and iron being almost a complement (over 96%) was used. Additionally, a trace content including carbon or other impurities (not exceeding tenths of a percent in overall) is also expected in this steel. The specific density of steel was 7650 kg/m^3^. Measurements were made for 16 angles at the location on the sample depicted with the letter A. The Figure 1 shows the view of the sample as well as the visualization of the first 5 measurement steps and rolling direction RD. The axis of first measurement is along to the transverse direction (TD) which corresponds to hard magnetization axis. The transducer was rotated by 22.5° after each measurement until reaching a full turn of 360°. In order to perform the measurement, the MBN system controlled by a personal computer (PC) equipped with a data acquisition board DAQ (NI USB-6251) was used. The system has been described in detail in paper [32]. The scheme of the measuring system setup is shown in Figure 2.

During the measurements, an MBN transducer having two sections was used (Figure 2c): the magnetizing and measuring ones. The magnetizing section consists of a coil wound on a C-shaped ferrite core. For the need of MBN signal sensing, a setup of coils wound on a rod core placed between the pole pieces of the electromagnetic yoke was utilized. In addition, a coil wound on one of the columns of the electromagnet and a Hall sensor located between its columns were used to control the magnetizing field conditions (magnetic induction and tangential component of magnetic field strength). For the needs of excitation, the sinusoidal waveform was generated using a digital-to-analog D/A converter of a DAQ board and then amplified using Power Amplifier (PA–OPA549) before driving the magnetizing coil. The excitation current frequency was set to 10 Hz, and the amplitude of the current was kept constant during the measurements. The estimated value of the magnetizing filed strength was 1.8 kA/m. The detailed discussion of the measuring conditions and analysis of the influence of the excitation field parameters on the MBN *TF* characteristic, as well as the choice of utilized settings (applied also in this paper) were presented and discussed elsewhere [24]. The MBN signal (*U*_BN_) induced in measuring coils was fed to the input of the analog processing system (APS) unit [24,32]. First, it was bandpass filtered in 0.6–97.5 kHz range and then amplified. The measurement was made in the angular steps, each time acquiring signals for the successive 10 periods of the magnetization process. The measurement was repeated 10 times, which made it possible to average the obtained results and minimalize the influence of interfering factors. Signal acquisition was carried out using the analog-to-digital A/D converter of DAQ board at sampling frequency of 250 kHz. Then, all measured signals were further processed using digital signal processing procedures.

## 3. The Time-Frequency Based Procedure for Evaluation of Easy Magnetization Axis

**Figure 3:**
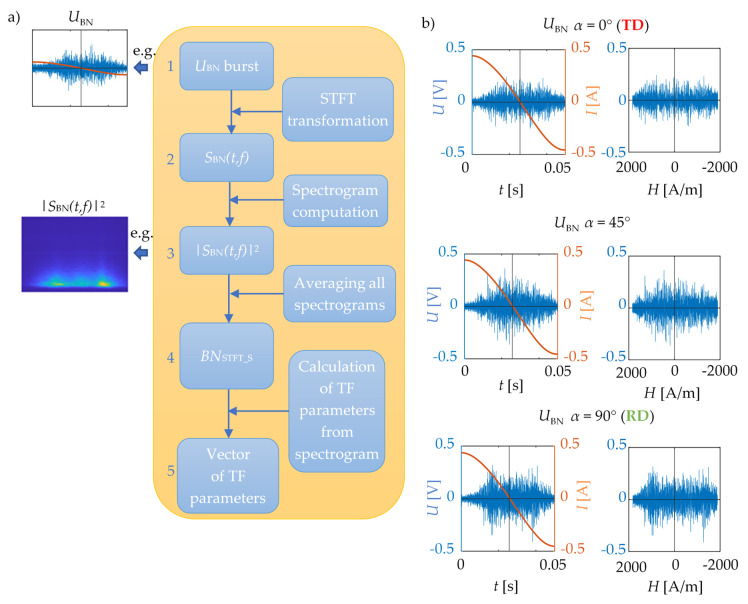
Time-frequency (TF) calculation procedure: (**a**) diagram of the procedure, (**b**) exemplary U_BN_ signals for orientation α = 0° (transverse direction, TD), α = 45°, and α = 90° (rolling direction, RD) vs. time (left column) and magnetic field (right column—presented in accordance to descending half of magnetizing period).

Figure 3 presents the successive stages of the procedure for evaluation of magnetic anisotropy based on the *TF* representation of the measured MBN signals along with exemplary MBN signals received for three different transducer’s orientations. The early step is to perform the initial signal processing and determine the *TF* representation.

First, in order to minimize low-frequency instrumentation disturbances, the digital signal filtration was performed by the Butterworth high pass filter (with a cutoff frequency *f*_C_ of 2 kHz). Then the measurements obtained were divided into half periods of magnetization, each corresponding to the individual MBN burst. In the next step, for each individual burst, transformation of the *U*_BN_ (time representation) to the *TF* domain was performed. For this purpose, the STFT transformation was used, which enables a homogeneous division of the computational grid in the *TF* space to be obtained and, in consequence, the dynamics of the Barkhausen phenomenon to be observed in detail [24]. The Kaiser type of the computational window having a length of 512 samples along with the overlapping technique with a rate of 0.75 was used during the STFT transformation. The given above parameters of the window resulted in a computational time step ∆*T* of 512 μs and frequency step ∆*F* of 488 Hz, what allowed to precisely observe the changes in dynamics of the MBN activity for various measurement angles. A more detailed analysis of the impact of the window width and the value of the filter’s cutoff frequency *f*_C_ on the quality of the acquired angular characteristics and the possibility of assessing the examined dependence is presented in the following sections of this paper. Finally, as a result, the complex *TF* representations *S*_BN_(*t*, *f*) of *U*_BN_ voltage signals were obtained. In next step, the spectrograms expressed as |*S*_BN_(*t*, *f*)|^2^ were calculated.

**Figure 4:**
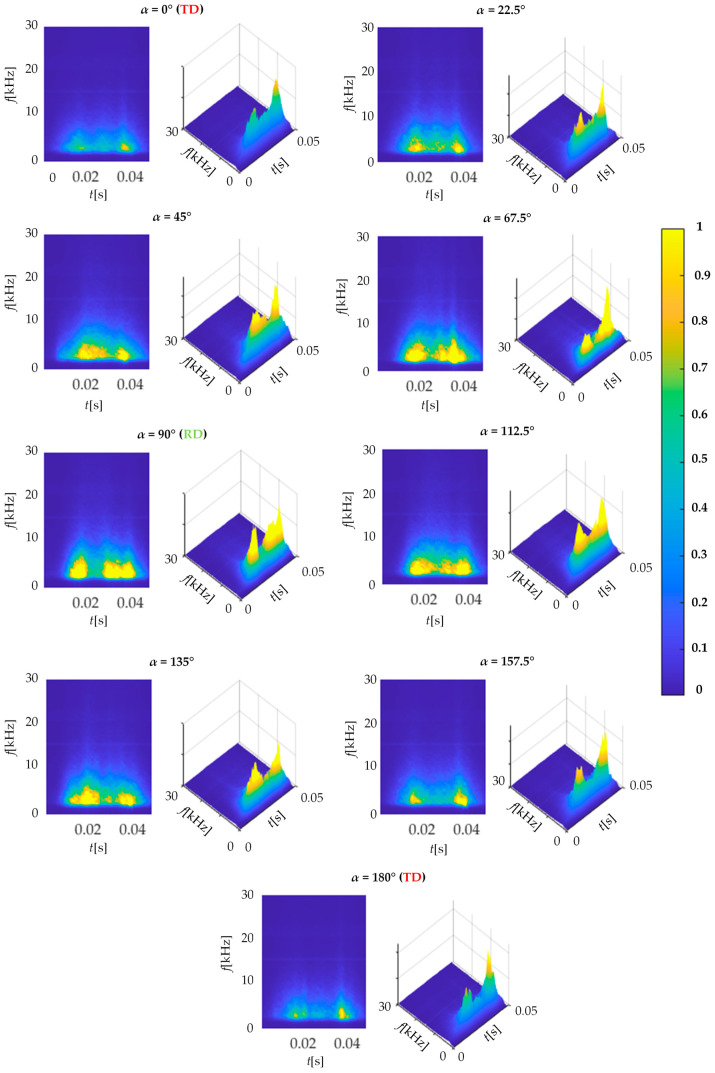
View of spectrogram activity in angle range between 0–180 degrees.

Multiple measurements for a single angular orientation enabled implementation of a smoothing procedure of the results obtained. Its purpose was to average all the spectrograms achieved for a single angular orientation of the transducer. Selected averaged spectrograms *BN*_STFT_S_ calculated for the measurements acquired within the half range of the transducer rotation (with respect to the TD from 0° to 180°) are shown in Figure 4. There is a noticeable difference between spectrograms for subsequent angles in the activity of the measured MBN. The highest activity was obtained for orientation of the transducer in accordance with the rolling direction of steel and along its easy magnetization axis (Figure 4, *α* = 90° i.e., RD). In this case, the highest energy level is visible practically throughout the entire MBN period, excluding the time span in which the magnetizing field changes its direction (around a time of 25 ms, see Figure 3b). At the same time, the frequency band occupied by the highest activity level of MBN is also the widest. Moreover, it can be seen that along with the gradual rotation of the transducer into the transverse direction to the RD (TD direction: *α* = 0° and *α* = 180°), a gradual delay of the beginning of the MBN activity area (arises more slowly) as well as the general decrease in its area can be seen. The bandwidth of the MBN activity is also noticeably reduced. On all the distributions presented, regardless of the angle of measurement, one can notice two distinct areas of MBN activity, one at the beginning of the period around 15 ms and the other at its end around 40 ms. In addition, a third area of activity is strongly visible at the RD angle. This follows the observations presented by other researchers. Considering this, those activity regions could be associated with the aforementioned (three) processes of nucleation of reverse domains, 180° and 90° DWs motion and the growth of the MBN activity for RD could be explained in reference to the large quantity of 180° DWs [2,25,28,30,31,33]. Despite clearly visible variation of the MBN activity in spectrograms, for proper assessment it is necessary to carry out a detailed quantitative analysis enabling the quantification of observed relationships.

The magnetic anisotropy of the material ends up with differences in the magnetic properties occurring for different test angles. Based on the spectrograms, it can be noticed that these changes in properties are then reflected in the course and the intensity of the observed Barkhausen phenomenon. In consequence, the dynamics of the energy distribution, concentration, centroid shift in time and frequency, and the degree of order/disorder or scope of changes of the MBN *TF* representation may be affected. Therefore, in order to quantify information expressing the angular variations of magnetic characteristics, a multi-parameter extraction was used to define vectors of *TF* features for each test angle. As a result, the set of several parameters carried by the *TF* representation were calculated from the *BN*_STFT_S_. The first group refers to some statistical properties, including various forms of mean values (i.e., arithmetic, geometric, etc.), centroid, variance or standard deviation, skewness or kurtosis. The next subset are the features describing of the shape of the *TF* spectrogram and its energy distribution or entropy, thus allowing the dynamics of variance, uniformity of distribution or the degree of disorder of the MBN spectral content to be assessed. The last, but no less important part of the *TF* features vector is parameters that indicate different characteristics values of the *BN*_TF_S_, such as: symmetry, center shift in the *t* or *f* axis, flatness, homogeneity or monotonicity, etc. The definition and properties of all proposed *TF* features and the used calculation procedure have been introduced earlier and discussed in detail in [32]. The results of the angular distributions of selected features will be presented in the following chapter.

## 4. Angular Distribution Results of Time-Frequency (TF) Features

**Figure 5:**
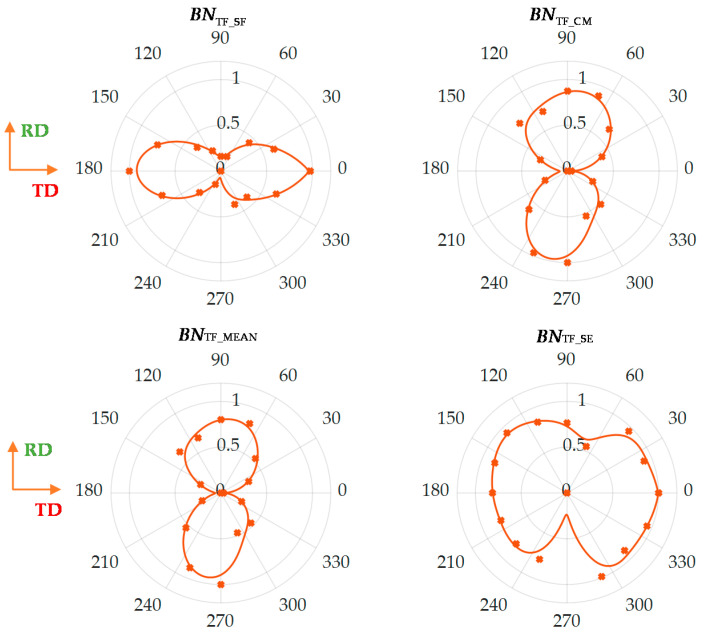
View of calculated parameters for 2 kHz and 512 window size: Markers represents the features values obtained for selected test angles; solid line refers the piecewise fitting result; all results are normalized with respect to maximum value.

Figure 5 presents results of four selected parameters obtained from the *TF* representation: spectral flatness *BN*_TF_SF_, concentration measures *BN*_TF_CM_, spectral entropy *BN*_TF_SE_ and mean value *BN*_TF_MEAN_ of the spectrogram. The chosen parameters refer to all three groups described above, allowing generalization of the *TF* characteristic. The definitions of the presented parameters were shown in Table 1. Before presentation all features were normalized, to eliminate the isotropic part of information (presented in 0–1 scale).

**Figure 6:**
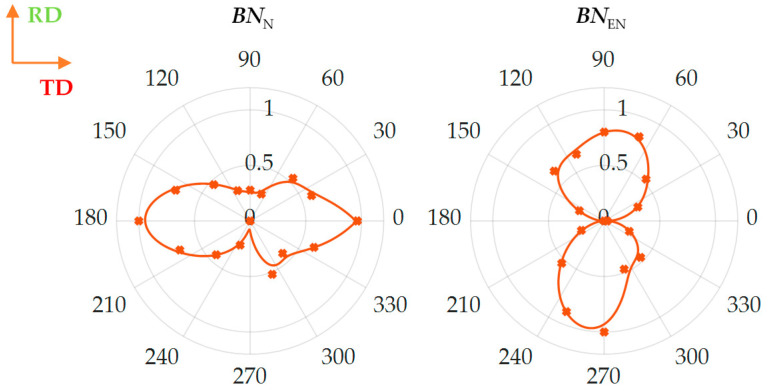
Results of the angular distributions of classical magnetic Barkhausen noise (MBN) parameters, that is the number of events BN_N_ and the energy BN_EN_ derived from time domain representation: markers represents the features values obtained for selected test angles; solid line refers the piecewise fitting result; all results are normalized with respect to maximum value.

All feature distributions were approximated using a piecewise fitted curve to facilitate the analysis and interpretation of the obtained angular characteristic. It should be emphasized that obviously interpreting the distributions obtained is not the same in each case and the minimum values of some presented features do not have to refer to the minimum values of others, and the same for maximums. First, two of the selected parameters relate to the assessment of the energy concentration of the analyzed spectrogram. The *BN*_TF_SF_ parameter specifies the ratio of the geometric to the arithmetic mean and obtains higher values when the distribution is homogeneous (e.g., random). In reference to the presented *TF* spectrograms, this parameter takes the highest values for the hard magnetization axis. As the transducer orientation approaches to 90 degree angle (easy magnetization axis), value of the parameter begins to decrease, which indicates a growth of MBN activity (energy) area. Confirmation of this observation is found in the distribution of the second parameter, the *BN*_TF_CM_, which takes higher values in case of the evenly distributed energy over the entire *TF* plane. However, at the same time, the parameter is not sensitive to small quantities. Thus when the level of MBN activity is growing in all spectral bands in general, the parameter value is reaching higher values as well. According to the received angular distribution, the *BN*_TF_CM_ value increases as the angle increases within the 0–90 degrees range. This parameter achieves the largest value in the direction of the easy magnetization axis, which is consistent with the observation for the angular distribution of the *BN*_TF_SF_ parameter. The spectral entropy allows the rate of disorder of the spectrogram to be assessed. It can be noticed that *BN*_TF_SE_ assumes the highest value for relatively wide orientation range around TD (*α* = 0° and *α* = 180°), at the same time showing a sudden decrease of value for the angle close to RD (*α* = 90° and *α* = 270°). This can be understood as leading the spectral distribution *BN*_TF_S_ to a higher degree of order and to accommodate the existing energy states for easy magnetization direction. The last presented parameter, *BN*_TF_MEAN,_ relates to the statistical quantity. The angular characteristic of the feature is well correlated with the course of the *BN*_TF_CM_. This confirms the earlier observations regarding the increase in value levels practically throughout the whole spectrogram space for angles consistent with the RD direction or close to it. All obtained distributions allow to draw similar conclusions. One can see a general indication of the directions of high and low activity of MBN, which are also consistent with the direction of, respectively, easy and hard magnetization. In reference to Figure 4, for the RD angle, one can observe a clear increase in MBN activity, expressed by a global increase in its level (reflecting among others, in *BN*_TF_MEAN_ and *BN*_TF_SF_ values). At the same time, the greatest energy values accumulate within the three mentioned sub-periods (affecting among others, the *BN*_TF_CM_), with a growing difference between areas with low and high MBN activity (accommodation of existing energy states depicted by among others, *BN*_TF_SE_). Moreover, this increase in activity is most visible on the presented spectrograms near the central part of the entire MBN signal period. From extensive research reported in the literature, RMA has a particular impact on activity in this area [25,30,31]. This would indicate the greatest impact of RMA on the distribution of resultant anisotropy and the occurrence of the easy magnetization axis. The information contained in the proposed *TF* features can be validated by classical MBN features. Figure 6 presents the distribution of two frequently utilized MBN parameters, i.e., number of events *BN*_N_ and energy *BN*_EN_. The distributions obtained confirm the directional properties of the tested steel, showing a clear increase in energy for the direction consistent with RD. An increase in activity for the RD direction was also observed in other publications [26,28], where authors explained the higher MBN activity (determined by the higher amplitude of the MBN envelope and RMS or energy values) by a much larger number of 180° domain walls in this direction. Similar conclusions were also presented in a number of other works [25,27,30,31], under consideration of only a middle part of the MBN signal associated with the movement of the 180° DWs and related to the influence of RMA. Based on the obtained angular characteristics (Figure 5 and Figure 6) one can notice good agreement with the presented *TF*-based results. Recently the authors presented detailed comparison of the various features obtained from the time, frequency and time-frequency domain [32]. A good correlation between the spectral flatness and number of events, and between concentration measure and energy as well was reported. The presented results in Figure 5 and Figure 6 confirmed the previous observations. The observed increase for RD in activity also translates into energy carried by MBN, while the decreasing number of events can be explained by the increase in the MBN phenomenon intensity and the superposition of smaller impulses into a larger cluster. This confirms the observations made for the parameters of the *TF* characteristics. Higher pulse values obtained for the RD direction compared to the TD direction cause a significant increase in energy value, but also a noticeable increase in the bandwidth occupied by the areas of highest activity, which (considering the scale of both factors) finally affects an increase in the value of the *BN*_TF_CM_ parameter. Furthermore, the increase in the difference between the energy states of the highest activity areas and the rest of the spectrogram determines the decrease in the *BN*_TF_SF_ parameter value. In addition, the overlapping of MBN events and accumulation of energy lead to a decrease in the parameter *BN*_TF_SE_. Considering all aspects, the results obtained indicated the possibility of using this proposed method based on the *TF* representation of full-period for the analysis of the resultant anisotropy in SiFe steel.

Finally, despite the sparse measurements (22.5° angular step) and the occurrence in some cases of non-compliance of measuring points with angular characteristics, which also affects the achieved approximations, the RD and TD axes can in almost all cases be uniquely identified by the location of maximum or minimum values of individual parameters.

However, due to the observed discrepancies, it becomes justified to verify the repeatability of the results obtained. As a significant number of MBN bursts were registered each time (10 measurements, 10 magnetization periods each) the assessment of the divergence of individual *TF* features obtained for subsequent measurement angles can be performed. Therefore, in the next stage of the work, the impact of possible causes affecting the range of the dispersion of parameter values obtained for a single angular orientation of the transducer was analyzed.

## 5. Analysis of the *TF* Features Dispersion

Several factors may influence the dispersion of the parameters’ distributions described above and in the previous section. They concern both the stage of measurement and hardware configuration of the system as well as the subsequent stage of data processing and the selection of computational procedure parameters. It should be emphasized that one of the key factors affecting the smoothness of the characteristic transitions between successive measurement angles is the number of adopted angular steps. The step applied in the paper is relatively large, however, it still makes it possible to determine general trends of the characteristic. On the other hand, it can be noted that errors for individual measurement points have a significant impact on the local course of the characteristic. Another key factor affecting errors is the relatively large size of the transducer used, which means that the measured values should not be treated as spot, but measurements in a certain vicinity of the given point. Furthermore, the minimization of possible influence of random factors on the recorded signals was obtained by repeating the measurements sequence several times under the same conditions (excitation parameters, transducer orientation angle in relation to the sample axis) and then by implementing the procedure of data averaging. At the same time, the influence of alternating interfering components having a frequency constant over time (if appears) would result in constant energy level over the entire spectrogram period for a given frequency band, and thus could also be clearly identified on the time-frequency distributions. Some systematic error may also be the result of a small angular shift between the sample edges and the actual magnetization direction, but it is replicated for all orientations in very similar scale and has no major impact on values dispersion or on the achieved shape of the distributions, but possibly only on the angular shift of the characteristics. Another possible source of measurement errors may also be a small misalignment of the transducer’s symmetry axis in relation to its rotation axis. This misalignment error would result in an asymmetrical angular relationship of the calculated *TF* parameters. However, it would not affect the assessment of the direction of the easy or hard magnetization axis, but would end in obtaining a different scale of values on both sides (assuming high repeatability of the angular magnetic properties of the steel the difference would be negligible) of the symmetry of *TF* features’ characteristics. Nevertheless, it would also not results in dispersion variation between orientations of the *TF* parameters. Therefore, bearing in mind the above considerations, the assessment of the impact of the computational procedure configuration on the repeatability and dispersion of the results still remains. There are two key elements within this aspect. The first concerns the choice of window width used during the transformation of data into the *TF* domain. The second is related to the pre-transformation data preparation stage and refers to the adjustment of the digital high-pass filter (HPF) band *f*_C_. These parameters are crucial in the process of obtaining information.

The window size affects the resolution of time and frequency steps of the computed *TF* representation during the STFT transformation. For a narrow window, a high resolution in the time domain and a lower one in the frequency domain is achieved. In the opposite case, the wider the window is, the lower the resolution in time (larger time steps) and the higher in frequency is achieved. Therefore, the adjustment of the size is crucial to sensitivity for variations in analyzed spectrograms. As discussed in the introduction, anisotropy can be caused by a number of factors affecting the various stages of the reorganization of the domain structure during a single period of magnetization, and consequently appearing with different activity of the Barkhausen phenomenon in the time sequences. Thus, when analyzing individual factors, there is a need to obtain a high resolution over time, enabling unambiguous distinction between subperiods of increased Barkhausen phenomenon activity. Recently, the authors have considered the possibility of using *TF* analysis to distinguish time spans of MBN activity corresponding to various factors affecting anisotropy [24]. In that work, the key aspect was to maintain high resolution over time. Therefore, a relatively narrow window (guaranteeing steps ∆*T* = 128 μs and ∆*F* = 1.952 kHz) was used during STFT transformation. However, the purpose of this work is to explore the possibility of conducting a quick assessment of the resultant anisotropy. The entire period of MBN activity is then analyzed, not its subperiods. Therefore, in this situation, a high resolution over time is not so crucial, and higher resolution in frequency may have a greater impact on the effectiveness of the proposed analysis.

The second factor that may affect the amount of dispersion of features in a given measurement group is the value of the lower band *f*_C_ of the MBN signal frequency range. As can be noticed in Figure 4, the highest MBN activity is obtained within the lower frequency range of presented spectrograms *BN*_TF_S_. This means that the selection of the lower band value can be decisive for the *TF* parameter distributions obtained during the analysis. In this range, interference from measuring instrumentation can affect the signal. In addition, distortions of a frequency close to the lower range of the hardware filter may also occur. Therefore, it is crucial to properly filter the signal prior further analysis. The cutoff frequency *f*_C_ of the digital high-pass filter should be high enough to effectively cut off the effect of low-pass interference, but at the same time low enough not to lose information about the magnetic anisotropy of the material.

Finally, during the analysis of the impact of both factors on the spread of *TF* parameters, a series of calculations was carried out for four cases determined by two window sizes: 128 and 512 samples and two digital filter border frequencies: 0.5 kHz and 2 kHz. The choice of window width was made taking into account the resolution in time and frequency that they guarantee. In the first case, it is possible to obtain relatively small steps of ∆*T* = 128μs and large of ∆*F* = 1.952 kHz, while in the second one inversely, relatively large steps of ∆*T* = 512μs and small steps of ∆*F* = 488 Hz. In this way, it was possible to estimate the impact of the individual resolutions of the computational grid on the repeatability of the obtained distributions. When choosing the *f*_C_ value, the cutoff frequency of the hardware filter (0.6 kHz) was taken into account, as well as the values of the frequency steps for both computational grids. Therefore, finally the analysis was carried out for *f*_C_ less than 0.6 kHz and greater than 1.952 kHz, which allowed evaluation of the spread of *TF* parameters for two extreme settings.

**Figure 7:**
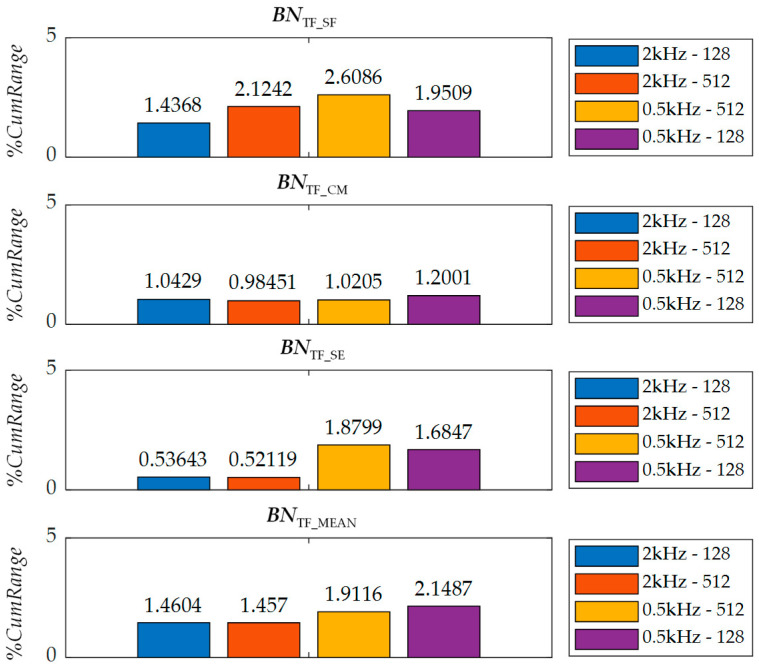
Bar graph of %CumRange indicator values of used features.

In the first stage of the analysis the averaged spectrograms *BN*_TF_S_ were calculated for each out of 10 measurements made for a given angular orientation. Next, for each averaged spectrogram the *TF* parameters were calculated. Then, spreads of *TF* features values were determined in reference to their average values achieved from all measurements. Finally, the values obtained for individual orientation angles (individual subsets) were further averaged and presented in the form of a common bar representation. Figure 7 presents a bar graph illustrating the dispersion range expressed in percentage scale (*%CumRange* indicator) of selected *TF* parameters accumulating the results achieved for all transducer orientations. Each bar specifies a dispersion range received under different data processing conditions. It can be noted that in most cases the smallest range value is obtained for the *f*_C_ of 2 kHz (first two columns of Figure 7). Considering the dispersion values of *BN*_TF_MEAN_, one can notice that when the *f*_C_ is 2 kHz the window size is not so significant, as the *%CumRange* values are comparable for both widths considered. However, the situation changes when the *f*_C_ is equal to 0.5 kHz. In that case, a smaller *%CumRange* is achieved for wider window. This confirms the high importance of the low-frequency range of the spectrogram on the *TF* features distributions. For a window width of 128 samples, the frequency step ∆*F* is 1.952 kHz. As a result, the use of a cutoff frequency of 0.5 kHz causes that any undesirable components of the transformed *U*_BN_ signal can be reflected in the lower range of the spectrogram band. Therefore, it can be concluded that the improvement of the robustness can be obtained when the *f*_C_ is set to greater value than the first frequency step of the calculation grid, i.e., for *f*_C_ = 2 kHz. On the other hand, in the case of window size of 512 sample the ∆*F* is equal to 488 Hz. Thus both considered cutoff frequencies are higher than the first frequency step of the spectrogram grid. Therefore, mostly similar results of the *%CumRange* are obtained for both *f*_C_ values, and the distributions of *BN*_TF_MEAN_ are more convergent in those cases.

In the case of the spectral flatness parameter, smaller spreads were obtained using a smaller size of window. However, as in the case of the mean parameter, also in this case the use of a higher cut-off frequency significantly reduces the dispersion range. In the case of the *BN*_TF_CM_ parameter, convergent results were obtained to those obtained for the *BN*_TF_MEAN_ parameter. The smallest spread value was obtained for a window of 512 samples and *f*_C_ of 2 kHz. However, it must be noted that the *BN*_TF_CM_ presents the highest stability over various computation parameters. The values of *%CumRange* obtained for feature *BN*_TF_SE_ are the smallest for window width 512 samples and *f*_C_ = 2 KHz. Thus, the *BN*_TF_CM_ feature proves high repeatability and resistance to interfering factors.

**Figure 8:**
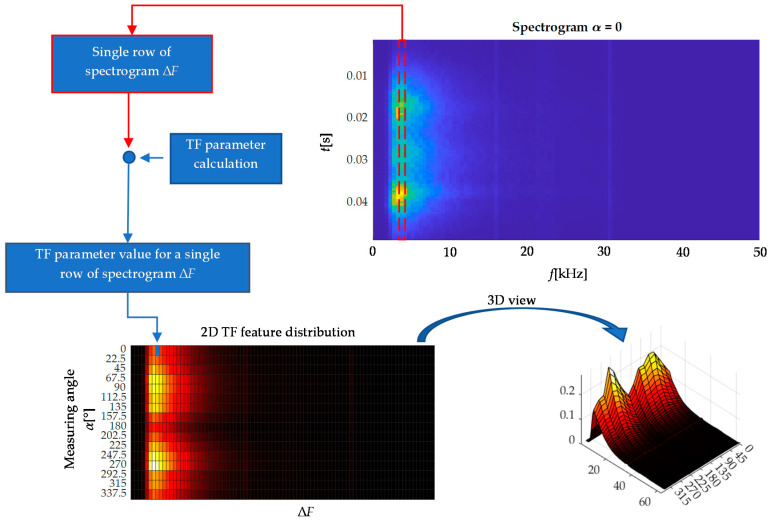
Diagram of the computational procedure of TF feature angular distribution in successive frequency ranges.

In order to further examine the influence of the cutoff frequency on the information contained in the lower frequency ranges of spectrograms, one more analysis was performed. Its purpose was to determine the angular distributions (characteristics) of *TF* parameters as a function of successive frequency ranges of the spectrogram’s computational grid. Graphic visualization of the calculation procedure is shown in Figure 8. For a given spectrogram, the entire time vector (single row) is considered, representing a single frequency range. Then the *TF* parameter value is calculated for this vector and the received value represents then a single cell of the resulting distribution presented in the form of the heat map.

**Figure 9:**
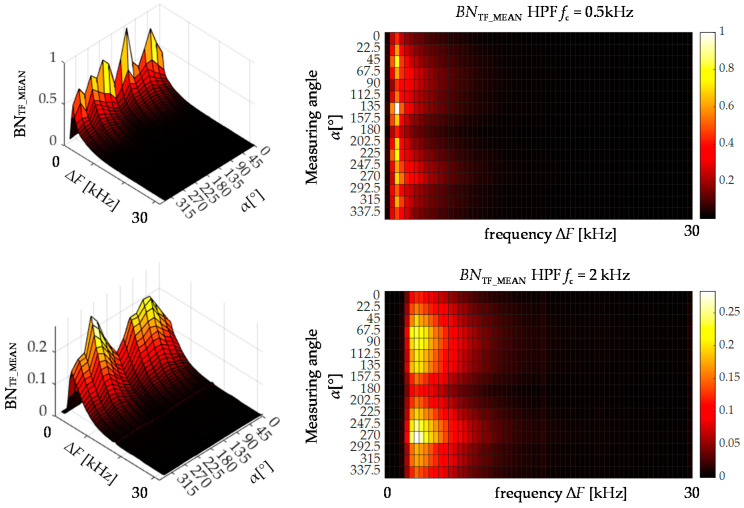
Visualization of the BN_TF_MEAN_ angular distribution in successive frequency ranges of the spectrogram; left column is a perspective presentation of the results in the right column.

Figure 9 presents two heat maps obtained for the *BN*_TF_MEAN_ parameter under two considered cutoff frequencies. In order to compare both conditions, the input spectrograms were computed using a window of 512 samples. Then the distributions were normalized to the (0–1) range and plotted in individual scales. If the lower frequency band value is used, the resulting distribution (top row) in the lower frequency ranges undergoes quite rapid changes of a rather random nature and not dependent on the angle of testing. Only for 90 and 270 degrees angles (along RD), in the higher frequency ranges, the characteristics present the angular relationship more visibly. In the second case, after applying a high-pass filter with a cutoff frequency of 2 kHz, a clear angular relationship in all compartments characterized by smooth and repeatable transitions between individual angles is obtained. Moreover, for angles that determine the axis of easy magnetization, large parameter values that at the same time cover a much wider frequency band are clearly visible. Attention should be paid to normalized ranges of values of both distributions as well. The unwanted components of low-frequency signals, not containing anisotropic information, reach much higher values. Therefore, they affect the sensitivity of the *TF* parameters and distort the crucial information.

Summarizing, the results of the analysis confirmed the impact of the computational parameters selection on the achieved dispersion of *TF* features values in successive subgroups, which in turn affects the repeatability of the entire method. In addition, it should be emphasized that the choice of the digital filter cutoff frequency *f*_C_ value is closely related to the window width value used during the STFT transformation. Therefore, in order to obtain the highest convergence of results and minimize the impact of external factors on parameter distribution, during the final analysis all results were (presented in previous chapter) achieved using the width of the window equal to 512 samples. This allowed the frequency resolution of spectrograms to be obtained enabling observation of even small variations of the MBN band, and at the same time to minimize the influence of the *f*_C_ value on the results. Nevertheless, aiming at maximum reduction of the influence of external factors on the computed *TF* distributions, additionally during the signal-processing procedure a cutoff frequency of 2 kHz was used. With these values of calculation parameters, the percentage dispersion of *TF* features values practically do not exceed 2%.

## 6. Conclusions

The ability to carry out rapid validation of the angular distribution of the magnetic properties of electrical steel is an important practical issue. Therefore, the purpose of this article was to investigate the possibility of using the Barkhausen effect and its time-frequency representation as a non-destructive tool to develop a procedure that allows retrieval of information about the resultant magnetic anisotropy in a classic grain-oriented electrical steel sheet. By assumption, this method is to be an alternative to the classic MBN approach, and ought to enable broad assessment of changes in the dynamics of the MBN phenomenon synchronously considering both time and frequency characteristics. For this purpose, tests were performed for a sample of 3% SiFe grain-oriented electrical steel. The measurements were made for 16 angles of transducer orientation, equally spaced within the range corresponding to full rotation. The paper presents the procedure of signal transformation into the *TF* domain, presentation of *TF* spectrograms and quantification of information contained therein. The obtained *TF* parameters expressing the change of the spectrogram allow the angle corresponding to the easy and hard magnetization axis to be determined. The possibility of extensive analysis of properties and observation of changes in the MBN activity makes this method possible to detect even small variations in the dynamics of the phenomenon over time resulting from the changes in magnetic properties. This creates a chance to obtain complementary information about the properties of the material, which can allow the formation of more complete knowledge.

The proposed method, based on the analysis of time-frequency characteristics in the full period of the MBN signal, allows detailed observation of the relationship between MBN properties expressed in time and in frequency for subsequent time moments. In consequence, it enabled the three characteristic MBN activity areas to be noticed. The occurrence of these areas in the MBN period has already been widely analyzed in many other works, and the angular characteristics of *TF* parameters obtained in this work are consistent with the other results reported. According to observations, these areas were associated with nucleation of reverse domains and, furthermore, the movement of the 180° and 90° DWs. The course of the first two was associated respectively with the magnetocrystalline anisotropy MCA and roll magnetic anisotropy RMA. Considering this, the angular distributions obtained by the authors underline the key importance of RMA for the alignment of resultant easy and hard magnetization axes in the tested steel. The angular characteristics of Barkhausen noise energy and numbers of events can be treated as confirmation of the effectiveness of the proposed *TF* method. These parameters, being used many times by other authors in the classic analysis, show convergent results with the *TF* ones. However, the results of both methods show some asymmetries and disturbances in the characteristics. The relatively small number of utilized measurement steps may have a decisive impact on the smoothness of the transitions of the characteristics obtained between successive measurement angles. The step applied in the paper makes it possible to determine general trends of the characteristic. On the other hand, errors for individual angles have a significant impact on the local course of the characteristics. Nevertheless, due to the sequencing of calculations procedures and the use of a sliding window over the analyzed signal, the STFT transformation leads to generalization of the phenomenon characteristics. In results, it can affect the increase of the robustness of the method for interfering external factors. This could be an explanation for obtaining slightly smoother courses of approximated *TF* characteristics.

In addition, the paper presents a detailed analysis of measurement errors and assesses the repeatability of the method in the context of the dispersion of *TF* parameters values within individual subsets of measurement signals. The choice of calculation parameters of the transformation procedure enables optimization of resolution in time and frequency, in reference to the nature of the observed changes. Based on the analysis conducted, it is crucial to emphasize the importance of the concentration measure parameter (carrying convergent information to MBN energy), which, regardless of the calculation parameters used, was characterized by high repeatability. The study also showed that the frequency value of the high-pass filter used for signal conditioning before *TF* analysis should not be lower than the value of the first step of the applied *TF* computational grid, which in turn allows for a significant increase in *TF* parameter robustness.

The presented method of time-frequency analysis is largely derived from the development of commonly applied methods of time or frequency analysis and is intended to present a possible path of development. The parameters defining the time-frequency characteristics are for the most part an extension of the classically used statistical coefficients. In principle, classical methods of analysis, in a single time or frequency domain, can be expressed as a generalization of the trends of changes presented in the time-frequency characteristics. Of course, at the same time it is not said that obtaining more detailed information will significantly improve the effectiveness of the MBN method in the future. However, the possibility of sequential data analysis itself (due to the potential to supplement the knowledge about the tested material) affects the validity of *TF* analysis usage and should be further examined. In the context of the presented arguments and already published papers, it can be seen that the use of time-frequency methods certainly does not affect the loss of information. On the contrary it can provide significant arguments to the discussion, as it gives a much wider perspective and brings a broader analysis of complex relationships of time and frequency characteristics.

In addition to the content of the work, one more aspect should be noted related to the development of future measurement systems. The classic method of analysis, despite many years of use, is still not standardized, and often the calculation procedures differ between researchers. The problematic element is, among others, the way of determining the MBN signal’s background level or procedure of counting the number of events. Therefore, the time-frequency calculation procedures are not showing greater complexity in terms of the practical implementation of the MBN method, especially currently when the advancement and miniaturization of computing units is common. It should be also considered that today’s diagnostic systems are increasingly based on monitoring many parameters and formulating multi-variate rules and correlation dependencies. Under these circumstances the results presented here show good potential. However the investigation with a large number of angular steps for steels having various textures and under influence of various anisotropy factors should be reaped. The issue, which also requires further research, is the synthesis method of the knowledge contained in *TF* representations, enabling greater efficiency to generalize and detailed analysis of information. The authors will present work on multiple samples and on multi-threaded analysis and synthesis procedures in the future.

## Figures and Tables

**Table 1:** Definition of selected features based on spectrogram *BN*_TF_S_ of *TF* representation *S*_BN_(*t*, *f*).

Feature	Formula
Spectral Flatness	$BN_{\mathrm{TF}\_\mathrm{SF}}=M\cdot N\frac{\prod_{i=1}^{N}\prod_{j=1}^{M}{\left|BN_{{\mathrm{TF}\_S}_{i,j}}\right|}^{\frac{1}{N\cdot M}}}{\sum_{i=1}^{N}\sum_{j=1}^{M}BN_{{\mathrm{TF}\_S}_{i,j}}}$
Concentration Measure	$BN_{\mathrm{TF}\_\mathrm{CM}}={\left(\sum_{i=1}^{N}\sum_{j=1}^{M}{\left|BN_{{\mathrm{TF}\_S}_{i,j}}\right|}^{\frac{1}{2}}\right)}^{2}$
Spectral Entropy	$BN_{\mathrm{TF}\_\mathrm{SE}}=\frac{1}{1-3}\log_{2}\sum_{i=1}^{N}\sum_{j=1}^{M}{\left(\frac{BN_{{\mathrm{TF}\_S}_{i,j}}}{\sum_{i=1}^{N}\sum_{j=1}^{M}BN_{{\mathrm{TF}\_S}_{i,j}}}\right)}^{3}$
Mean	$BN_{\mathrm{TF}\_\mathrm{MEAN}}=\frac{1}{N\cdot M}\sum_{i=1}^{N}\sum_{j=1}^{M}BN_{{\mathrm{TF}\_S}_{i,j}}$
